# Entropy-Based Registration of Point Clouds Using Terrestrial Laser Scanning and Smartphone GPS

**DOI:** 10.3390/s17010197

**Published:** 2017-01-20

**Authors:** Maolin Chen, Siying Wang, Mingwei Wang, Youchuan Wan, Peipei He

**Affiliations:** 1 School of Remote Sensing and Information Engineering, Wuhan University, Wuhan 430079, China; wmwscola@whu.edu.cn (M.W.); ychwan@whu.edu.cn (Y.W.); 2 Jiangsu Hi-Target Marine Technology Co., Ltd., Nanjin 210032, China; wangsiying@whu.edu.cn; 3 School of Resources and Environment, North China University of Water Resources and Electric Power, Zhengzhou 450046, China; hepei@ncwu.edu.cn

**Keywords:** terrestrial laser scanning, registration, sensor combination, point cloud, information entropy

## Abstract

Automatic registration of terrestrial laser scanning point clouds is a crucial but unresolved topic that is of great interest in many domains. This study combines terrestrial laser scanner with a smartphone for the coarse registration of leveled point clouds with small roll and pitch angles and height differences, which is a novel sensor combination mode for terrestrial laser scanning. The approximate distance between two neighboring scan positions is firstly calculated with smartphone GPS coordinates. Then, 2D distribution entropy is used to measure the distribution coherence between the two scans and search for the optimal initial transformation parameters. To this end, we propose a method called Iterative Minimum Entropy (IME) to correct initial transformation parameters based on two criteria: the difference between the average and minimum entropy and the deviation from the minimum entropy to the expected entropy. Finally, the presented method is evaluated using two data sets that contain tens of millions of points from panoramic and non-panoramic, vegetation-dominated and building-dominated cases and can achieve high accuracy and efficiency.

## 1. Introduction

The applications of terrestrial laser scanning (TLS) are continuously growing in areas such as city modeling, heritage documentation, manufacturing, and terrain surveying. The primary purpose of terrestrial laser scanning is to generate a complete surface model of the target object. However, because the limits of coverage vary and interruptions exist, a series of scans from different views are generally necessary. Hence, point clouds from various scans have their own coordinate frames. To obtain a complete model, point clouds from multiple scans must be transformed into a common uniform frame. This process is called registration.

Standard method for the registration task includes using artificial targets and semi-automatic registration. There are many types of artificial targets such as spheres [1] and planar targets [2], and such targets generally have special shapes or reflective features. When the targets are identified, transformation parameters can be calculated based on corresponding targets between different scans. One drawback of this method is that it takes too much additional time to arrange the targets in the scene. In some extreme conditions, it is impossible to place any targets. Additionally, placing artificial targets inevitably causes occlusions and disrupts the integrality of the data. Semi-automatic registration is also a commonly used registration method, which possesses a high universality and has been implemented in many commercial or opensource software packages (e.g., Riscan, Cyclone and Cloudcompare), and the corresponding points are picked manually to calculate the transformation parameters. Nevertheless, it sometimes takes much time and manpower when there are a number of scans.

To avoid the manual intervention, much research has been conducted focusing on automatic registration. Generally, automatic registration comprises two stages: coarse registration, roughly aligning scans and producing good initial pose parameters, and fine registration, obtaining final registration results with high precision. The most widely used method for fine registration is the Iterative Closest Point (ICP) algorithm introduced by Besl and McKay [3] and Chen and Medioni [4]. Transformation parameters between two scans are estimated iteratively until the sum of the squares of Euclidean distances between corresponding points converge to the minimum. Variants and optimizations have been proposed in various contexts since then, such as mathematical framework [5,6], corresponding metric [7,8], corresponding selection and weighting [9]. The drawback is that the distances will most likely converge to a local minimum without a good prior alignment. Therefore, methods to roughly align two original points in the coarse registration stage are important to the ICP algorithm.

A general line of thought for coarse registration is based on distinctive spatial elements, such as points, lines or planes. Those spatial elements generally have unique features, which are different from most others and can be extracted for correspondence searching. Scale Invariant Feature Transform (SIFT) [10] is one of the most widely used point features and can be classified as 2D key points. Bendels et al. [11] introduced SIFT for TLS points registration combing SIFT features from camera images with surface elements from range images. Barnea and Filin [12] proposed an autonomous model based on SIFT key points of panoramic images. This method addresses the registration of multiple scans. Application of the SIFT feature to reflectance images was introduced by Böhmand and Becker [13]. False matches caused by symmetry and self-similarity are filtered by checking the geometric relationship in a RANSAC filtering scheme. The SIFT feature was also used on reflectance images by Wang and Brenner [14] and Kang et al. [15,16]. Weinmann et al. [17] extracted characteristic feature points from reflectance images based on SIFT features and projected them into 3D space to calculate transformation parameters. This algorithm can achieve a high accuracy without fine registration by using 3D-to-3D geometric constraints. Besides SIFT descriptor, other image features also have been used, such as Moravec operator [18]. Methods mentioned above mainly rely on mature image processing algorithms which are efficient and convenient, but generally require large overlap areas to make sure enough correspondences. To relax the overlap requirement and adapt to the case of minimal overlap, Renaudin et al. utilized the linear features from photogrammetric and TLS dataset for the registration of multiple scans, with the coregistration of image and point cloud as a byproduct [19]. Similarly, photogrammetric linear and planar features were used for scan registration by Canaz and Habib [20], to avoid the requirement of large overlap.

In situations of strong viewpoint changes or poor intensity resolution, a method using 2D features is prone to failure; 3D features display more robust performances. Thus, many studies have focused on 3D point features for registration. Those methods extract 3D feature point sets and identify corresponding points to recover the transformation between two scans. Gelfand et al. [21] proposed an integral volume descriptor to detect feature points and match those points using an algorithm called branch-and-bound correspondence search. Theiler et al. [22] extracted Difference-of-Gaussian (DoG) and 3D Harris [23] key points from voxel-filtered datasets as input to the 4-Points Congruent Sets algorithm [24] to achieve coarse registration. Rusu et al. [25] estimated a set of 16D features called Point Feature Histograms (PFH), which are robust to scale and noise, providing good starting points for ICP. Then, Rusu et al. [26] applied some optimizations to PFH and proposed Fast Point Feature Histograms (FPFH) reducing the computation time dramatically. Examples of point features also include normal vector [27], distance between target point and center of neighboring points [28], 2.5D SIFT [29], curvature [30] and rotational projection statistics feature [31]. Stamos and Leordeanu extracted the intersection lines of neighboring planes as the primitives and calculated the transformation parameters with at least two line pairs [32]. Yao et al. presented a common framework for the automatic registration of two scans with linear or planar features and the orientation angles and distances were used to find candidate matches [33]. Yang and Zang used curves as matching primitives to find the initial transformation for point clouds with freeform surfaces, such as statues and artifacts [34]. Planes are also used for coarse registration in many studies. Theiler and Schindler [35] used intersecting planes to generate a set of virtual tie points, which are described by properties of corresponding parent planes. Then, tie points matching is guided by those descriptors. In the work of Dold and Brenner [36], planar patches were described by features including area, boundary length, bounding box and mean intensity value and matched with the help of image information. In this approach, those features of planar patches are sensitive to density and occlusions; thus, Brenner et al. [37] proposed a more robust method with planar patches. Plane triples were used instead of single patch in the matching process based on a sensible criterion. Pu et al. [38] combined the semantic features of planar patches and GPS position to derive the mathematical formulation of transformation parameters. The semantic information was also used in [39], in which Yang et al. detected features points based on semantic feature and the matching was processed by searching for corresponding triangles constructed and eliminate from the feature points. Kelbe et al. [40] calculated the transformation parameters for the forest TLS data based on the results of tree detection, which can be obtained from some previous work [41,42]. Some other geometric elements are also used in the registration, such as salient directions [43], cylindrical and polygonal objects [44], fitted objects in industrial environments [7] and other fitted geometric primitives [45]. To identify a good feature combination, Urban and Weinmann [46] presented a framework to evaluate different detector-descriptor combinations, in which five approaches are involved.

Another train of thought depends on external sensors (e.g., GPS/IMU) to record the position and orientation of each scan. Point clouds from different scans can be registered easily. This method is often used in mobile laser scanning [47,48] but can also be used in terrestrial laser scanning [38,49]. External sensors are helpful for registration of terrestrial point clouds, although the high cost of external sensors limits the application of this method.

The registration techniques described above suggest that most methods focus on detailed information extracted from point clouds to achieve the registration task. In complex scenes, feature extraction and matching are time-consuming and prone to failure if too much symmetry, self-similarity and occlusions exist. As the external sensors are quite helpful for point registration, this paper presents a novel method for the automatic coarse registration of leveled point clouds, combining terrestrial laser scanner with the smartphone, which is low-cost compared with professional sensors. This method works without synchronization between scanner and smartphone and jumps out of detailed features to register terrestrial point clouds from a macroscopic perspective. Scanner positions are roughly measured by the smartphone GPS and the distance between neighboring scanner positions is used as a translation constraint. The distribution coherence of the whole points from two scans is measured by 2D distribution entropy and used to identify optimal transformation parameters. The main contributions of this paper are as follows:

- combining the terrestrial laser scanner with smartphone for coarse registration;
- using 2D projection entropy to measure the distribution coherence between two scans; and
- presenting the Iterative Minimum Entropy (IME) algorithm to correct initial transformation parameters and reduce the effect of positioning error from the smartphone GPS.

## 2. Methodology

### 2.1. Combining the Terrestrial Laser Scanner with Smartphone

Smartphones are currently commonly used and quite popular in daily life. Most smartphones have the GPS and have previously been utilized in the processing of point clouds [50,51]. The position of a laser scanner can be measured by the GPS on a smartphone by an SDK of network maps such as Google Map or phone applications such as GPS checker, etc. To measure the scan position, a smartphone can easily be attached to the scanner when GPS data are being collected; this process does not produce a highly accurate measurement of scanner position, but is rapid and convenient.

**Figure 1:**
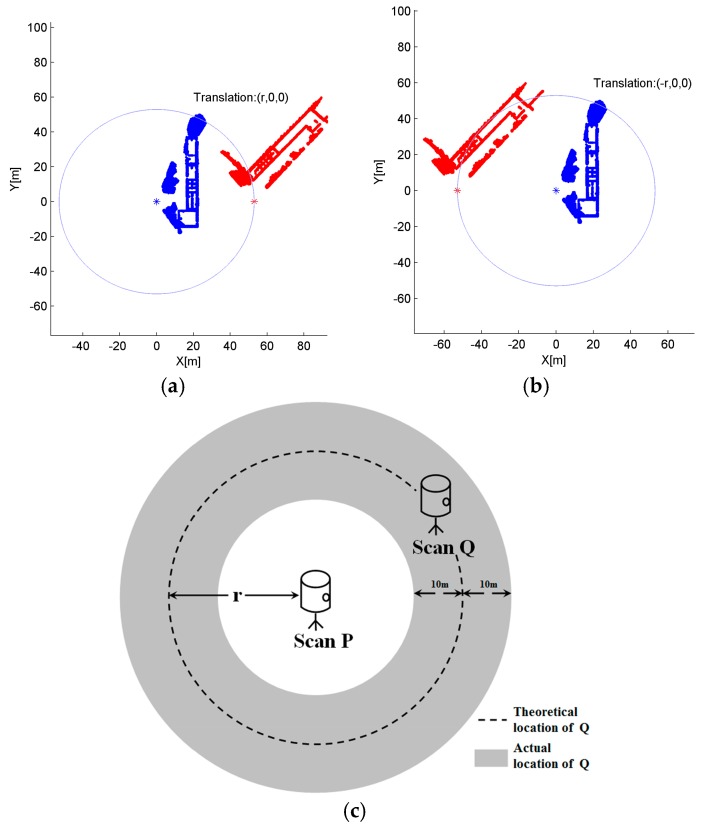
Relation between P and Q: (**a**,**b**) two different postures of P and Q with the same distance r, and the distance r is not enough to recover the transformation between them; and (**c**) the uncertainty of Q’s location.

With each scan position measured, the distance between adjacent scans can be obtained. The distance measuring with smartphone GPS does not require the intervisibility between neighboring scans, bringing a favorable flexibility for the scanner set up, which is applicable to most cases, especially in the plot containing many trees or other obstacles. For two scans, P and Q, distance r between their scan positions can then be calculated. With r, it is possible to determine the relative position relation between the origins of P and Q. If P is set reference whose origin coordinate is (0, 0, 0) under its own coordinate system, then Q’s scan position will be located on the circle whose center is P and radius is r. However, because the framework of TLS data is usually related with the orientation of the scanner, e.g., the x-axis is commonly the direction of the starting laser beam on the scanner’s rotating plane, y-axis is set to the direction perpendicular to x-axis whirling counterclockwise and the direction of z-axis follows the right-hand rule in Reigl VZ-400 laser scanner used in this paper, the posture of the point cloud is thus unknown without external reference. Although the attitude parameters can be obtained by the gyroscopes, it is also required to know how the scanner constructs the frame to associate the postures for two scans, which will bring more manual intervention and make the method poor in the applicability for the data from unknown scanner. The distance r itself is not enough to recover the transformation between two scans directly, as there are many possible distributions for P and Q only with the constraint r, which is shown in Figure 1a,b. Nevertheless, the most appropriate transformation parameters can still be searched based on the spatial relation between scans P and Q, with postures considered. The problem is that smartphone GPS commonly has a location error up to 10 m, causing an error which may reach 20 m in the distance r, resulting in more uncertainty for the location of Q (as shown in Figure 1). We propose an Iterative Minimum Entropy (IME) algorithm to reduce the effect of locating error and refine the initial transformation parameters in Section 2.2.3.

### 2.2. Registration with 2D Projection Entropy

#### 2.2.1. Searching for the Minimum Entropy in 3D Space

Information entropy [52] is the measurement of the expected value of information and represents the uncertainty of an event, which has been used in many areas, such as remote sensing image evaluation [53], classification [54] and image matching [55]. This section exploits information entropy to measure the distribution coherence of point clouds from two neighboring scans.

According to information theory, for a discrete random event $X=\{X_{1},X_{2}\ldots X_{n}\}$ with corresponding probabilities $P^{\prime }=\{p_{1},p_{2}\ldots p_{n}\}$, the amount of information of *X* can be measured by

(1) $$H\left(X\right)=H\left(p_{1},p_{1},\ldots p_{n}\right)=-\sum_{i=1}^{n}p_{i}\;log\;p_{i}.$$

The more information *X* contains, the larger H(X) will be, with more uncertainty of *X*.

For two scans P and Q, different spatial distributions achieved by different transformation parameters represent different levels of point distribution uncertainty between two scans. The uncertainty of the point distribution can also be measured by entropy. As the relative position relation between P and Q can be obtained through the smartphone in Section 2.1, P and Q can be transformed into the uniform coordinate framework. In this paper, an r translation is applied to Q, where r is the coarse distance between origins of P and Q, and the origin of Q will turn into (r, 0, 0) under the coordinate framework of P. The space covered by P and Q is subdivided into a regular cuboid, and a cell C can be treated as an event. The probability that one point falls into C is

(2) $$p(C)=\frac{n(i,j,k)}{N},$$

where *i*, *j* and *k* are the index of C with n(*i*, *j*, *k*) points falling into and *N* is the total number of points in both P and Q. The distribution entropy of the whole points thus can be calculated by

(3) $$H\left(P,Q\right)=-\sum_{i=1}^{in}\sum_{j=1}^{jn}\sum_{k=1}^{kn}\frac{n(i,j,k)}{N}log\frac{n(i,j,k)}{N},$$

where *in*, *jn* and *kn* are the cell numbers along three axes x, y and z. In case of the same total number of points, higher *H(P,Q)* indicates more cells containing at least one point and more discrete distribution of these points. If all corresponding points in P and Q fall into the same cell, the entropy will generally achieve the minimum. If we get can identify the minimum entropy among different point distribution cases, the corresponding transformation parameters will most likely be an ideal answer. Thus, the task of registration can be transformed into finding the minimum distribution entropy of P and Q. 

**Figure 2:**
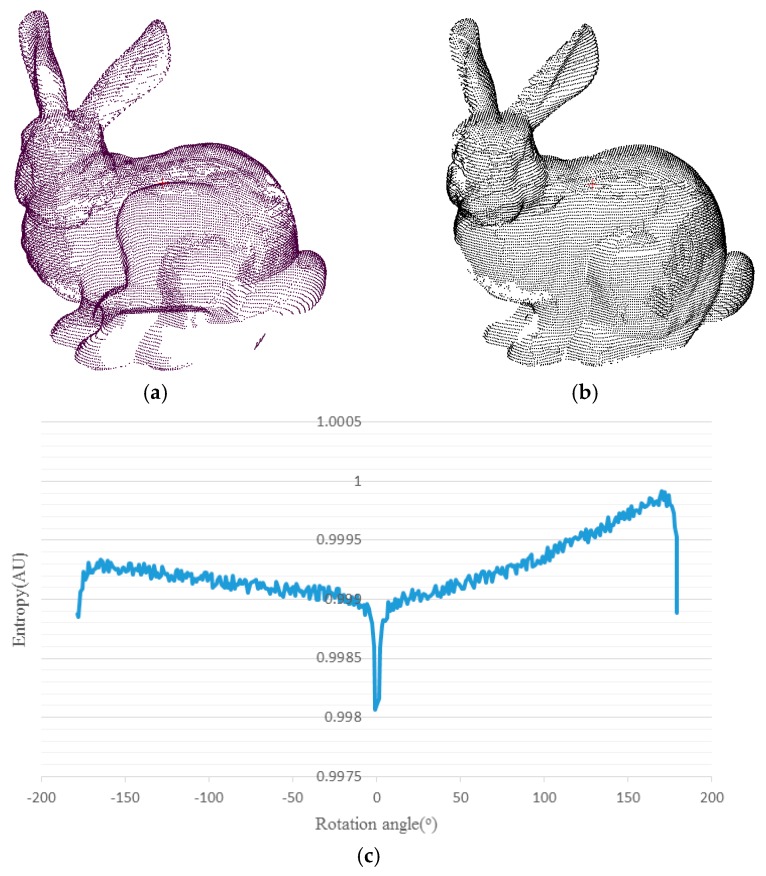
The relation between γ and its corresponding entropy: points in (**a**,**b**) are previously registered, and the entropy is calculated when points in (**b**) are being rotated γ degrees around the z axis. The entropy change with γ is recorded in (**c**).

Figure 2 shows the relation between rotation angle γ and the corresponding entropy in Stanford Buddy datasets in 3D space. Points in Figure 2a,b are previously registered, and γ is the rotation angle of points in Figure 2b around Z axis. Entropy corresponding to different γ is shown in Figure 2c. Figure 2c shows that entropy achieves the minimum when γ is 0°; thus, the optimal transformation parameters can be observed in the process of searching for minimum entropy. However, for point clouds captured in real scenes, there are always three rotation parameters that must be considered simultaneously, making the search process a three-nested loop, which is time-consuming. In addition, the cuboid partition is executed in a large-scale 3D space, also making this method inefficient. Thus, some changes are essential to simplify the search process, which will be described in next section.

#### 2.2.2. Transformation from 3D to 2D Space

To reduce the cost of searching process, we project points onto the plane of X-o-Y. On this plane, the 2D space is divided into a regular grid, with the number of points falling into every block recorded. Then, transformation parameters are detected by searching for the minimum distribution entropy on this grid.

Before projecting points onto X-o-Y plane, preprocessing is applied for the original points, including ground filtering and removing small clusters. The purpose of filtering out ground points and small point clusters is to reduce the spatial extent and number of points after projection because a discrete or flying point may provide an extreme coordinate value, expand grid extent and produce more redundant empty blocks. As described by Pirotti et al. [56], ground filtering is critical to the definition of above-ground elements, which are small point clusters in this paper. Vosselman and Mass [57] presented an overview of ground filtering methods that can be divided into three primary classes. Those three methods are based on progressive densification of a triangle mesh, mathematical morphology and linear prediction and hierarchic robust interpolation. Although point filtering is a wide field, we can narrow it to the case of our research, in which ground points of high accuracy are not required. Preprocessing is conducted in three major steps:

- Ground filtering: The ground is removed using progressive densification of a triangle mesh.
- Clustering above-ground points: The clustering process is based on distance. If the distance between p and its closest point q is within the distance threshold, then p and q will be identified as the same cluster.
- Removing small clusters: In this paper, a cluster is removed if the number of points in the cluster is less than 500.

**Figure 3:**
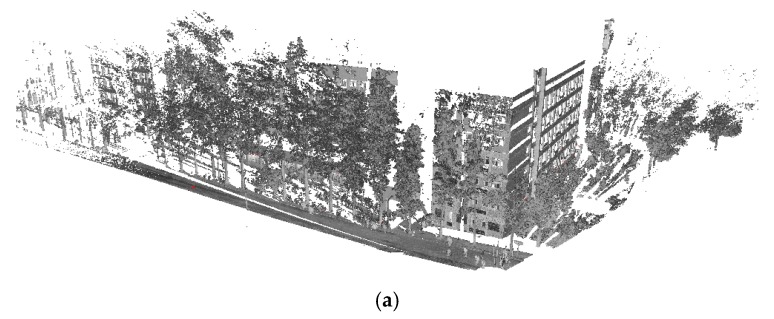

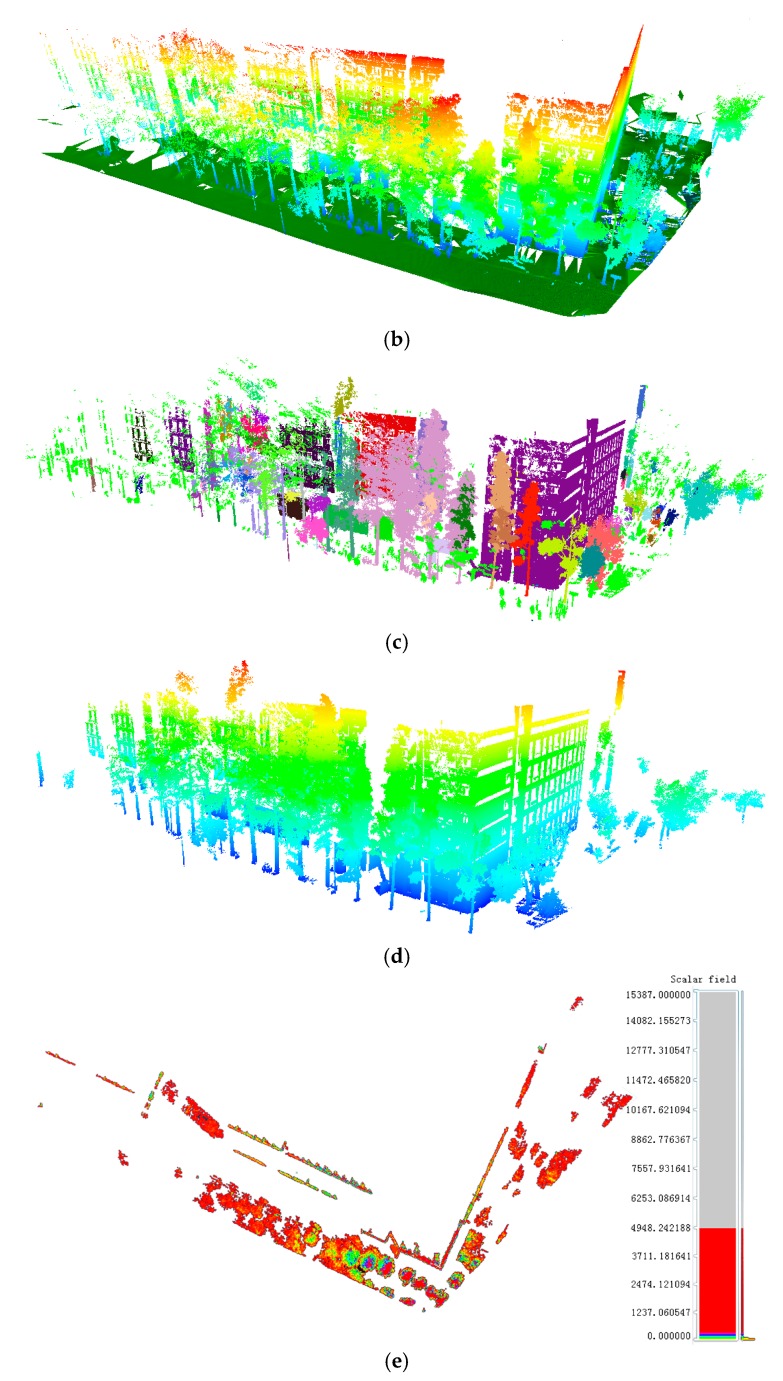
Transformation from 3D to 2D: (**a**) original points; (**b**) ground filtering; (**c**) clustering result with small clusters colored by green; (**d**) filtering result and the number of points is 3,407,526; and (**e**) projecting result and the number of points in each block is rendered by different colors of the block center. The number of points is 27,706.

The remaining points will be projected onto the X-o-Y plane, primarily comprising architectures, vegetation and some other types of points. Typically, we follow the assumption that a laser scanner is set approximately vertical during data acquisition [37]. Sometimes it is not the case, such as scanning a high building in close distance with a tilted scanner posture, and this can be compensated by rotating the point cloud through Principal Components Analysis (PCA), as described by Polewski et al. [58]. Thus, the coordinates x and y can be directly used as the projected coordinates on X-o-Y plane and Figure 3 shows an example of the projection in this paper. Then the scan Q can be transformed into P’s coordinate framework on X-o-Y plane, with the translation of distance r (obtained in Section 2.1) and the origin of Q will turn into (r, 0).

Under the same coordinate framework, the distribution entropy of P and Q can be calculated. The combined 2D space is subdivided into a regular grid of block size t_G_ to generate a grid G(P,Q). The size of G(P,Q) can be calculated by

(4) $$m_{G}=\left\lfloor \left(x_{max}-x_{min}\right)/t_{G}\right\rfloor +1,$$

(5) $$n_{G}=\left\lfloor \left(y_{max}-y_{min}\right)/t_{G}\right\rfloor +1,$$

where $x_{max}$, $x_{min}$, $y_{max}$ and $y_{min}$ represent the bounding rectangle of the projected points of both P and Q. The index of the block that point p falls into can be obtained using

(6) $$i=\left\lfloor \left(x_{p}-x_{min}\right)/t_{G}\right\rfloor +1,$$

(7) $$j=\left\lfloor \left(y_{p}-y_{min}\right)/t_{G}\right\rfloor +1,$$

where $x_{p}$ and $y_{p}$ are the projected coordinates of *p*. The distribution entropy for P and Q can be calculated on G(P,Q) with

(8) $$H(P,Q)=-\sum_{i=1}^{m_{G}}\sum_{j=1}^{n_{G}}\frac{n(i,j)}{N}log\frac{n(i,j)}{N},$$

where n(i,j) is the number of points falling into the block of index (*i*, *j*) and N is the total number of points in both P and Q.

Fundamentally, the Euler distance, which is generally used in ICP, can also be utilized to measure the distribution for two scans. However, the convergence basin of ICP is usually too small [20] and it works only with an accurate description for the postures of two scans. For two coincident wall surfaces, similar distance error will be obtained as the height of the wall changes. However, the entropy will change as the height of the wall changes, as the space distribution of all the points is considered. Additionally, computing the distance error related with ICP is more time consuming than the computation of entropy, making Euler distance less practical in the coarse alignment stage.

**Figure 4:**
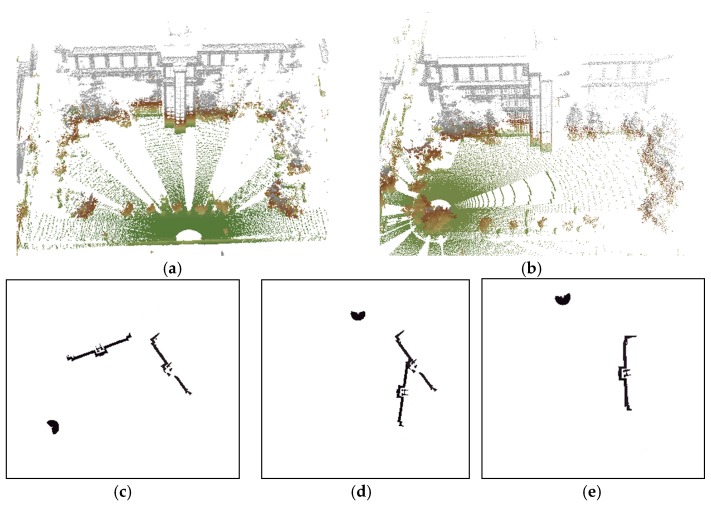
Search for the minimum entropy: (**a**,**b**) the original point clouds; (**c**,**d**) searching process; and (**e**) the postures corresponding to the minimum entropy.

After point projection, the registration task will convert to 2D point alignment and only the rotation angle around the z axis needs to be considered. With the origins of P and Q have been set (0, 0) and (r, 0) under P’s coordinate framework, the postures of the two scans are only related with the rotation angles around (0, 0) and (r, 0), respectively. The 2D relation between P and Q when registered will be

(9) $$\left[\begin{array}{cc} \cos(\kappa_{p}) & -\sin(\kappa_{p})\\ \sin(\kappa_{p}) & \cos(\kappa_{p}) \end{array}\right]\left[\begin{array}{c} x_{p}\\ y_{p} \end{array}\right]=\left[\begin{array}{cc} \cos(\kappa_{q}) & -\sin(\kappa_{q})\\ \sin(\kappa_{q}) & \cos(\kappa_{q}) \end{array}\right]\left[\begin{array}{c} x_{q}\\ y_{q} \end{array}\right]+\left[\begin{array}{c} r\\ 0 \end{array}\right],$$

where P and Q rotate angles of $\kappa_{p}$ and $\kappa_{q}$, respectively, around their own z axis. Thus, the 2D point alignment will convert to finding appropriate rotation parameters $\kappa_{p}^{\prime }$ and $\kappa_{q}^{\prime }$ to gain the minimum entropy:

(10) $$\left[\kappa_{p}^{\prime },\kappa_{q}^{\prime }]=\arg\;\min\;H_{r}(\kappa_{p},\kappa_{q}),\kappa_{p},\kappa_{q}\in [0,360^{\circ }\right],$$

where $H_{r}(\kappa_{p},\kappa_{q})$ is the distribution entropy under the rotation parameters $\kappa_{p}$ and $\kappa_{q}$ when the distance between P and Q is r. The coordinates of P and Q need recalculation every time $\kappa_{p}$ and $\kappa_{q}$ vary, which is time consuming. Thus, point cloud simplification is necessary and we achieve it by grid simplification in this paper. The point cloud from P or Q is assigned to a grid of block size d_G_ and every block is represented by its center, with the number of points falling into it recorded. Figure 4 illustrates a simple example of the searching process. Point clouds in Figure 4a,b rotate around their own origins, (0, 0) and (r, 0), and the continuous ranges of $\kappa_{p}$ and $\kappa_{q}$, which are [0,360°], are discretized with the interval of 1°. The rotation angles corresponding to the minimum entropy are selected to calculate the initial transformation parameters, as shown in Figure 4e.

With the assumption of the approximately vertical scanner, the z component of translation parameters between P and Q can be roughly determined based on the distance between the ground parts. Then, the relation between two registered scans P and Q can be extended back to 3D space by

(11) $$\left[\begin{array}{ccc} \cos(\kappa_{p}) & -\sin(\kappa_{p}) & 0\\ \sin(\kappa_{p}) & \cos(\kappa_{p}) & 0\\ 0 & 0 & 1 \end{array}\right]\left[\begin{array}{c} x_{p}\\ \begin{array}{l} y_{p}\\ z_{p} \end{array} \end{array}\right]=\left[\begin{array}{ccc} \cos(\kappa_{q}) & -\sin(\kappa_{q}) & 0\\ \sin(\kappa_{q}) & \cos(\kappa_{q}) & 0\\ 0 & 0 & 1 \end{array}\right]\left[\begin{array}{l} \begin{array}{c} x_{q}\\ y_{q} \end{array}\\ z_{q} \end{array}\right]+\left[\begin{array}{l} \begin{array}{c} r\\ 0 \end{array}\\ \Delta h \end{array}\right],$$

where $\kappa_{p}$ and $\kappa_{q}$ are rotation angles around the z axis for P and Q, r is the distance between the two scan positions of P and Q and Δh is the transformation along the z axis. The transformation from Q to P will be

(12) $$\left[\begin{array}{c} x_{p}\\ \begin{array}{l} y_{p}\\ z_{p} \end{array} \end{array}\right]={\left[\begin{array}{ccc} \cos(\kappa_{p}) & -\sin(\kappa_{p}) & 0\\ \sin(\kappa_{p}) & \cos(\kappa_{p}) & 0\\ 0 & 0 & 1 \end{array}\right]}^{-1}\left[\begin{array}{ccc} \cos(\kappa_{q}) & -\sin(\kappa_{q}) & 0\\ \sin(\kappa_{q}) & \cos(\kappa_{q}) & 0\\ 0 & 0 & 1 \end{array}\right]\left[\begin{array}{c} x_{q}\\ \begin{array}{l} y_{q}\\ z_{q} \end{array} \end{array}\right]+{\left[\begin{array}{ccc} \cos(\kappa_{p}) & -\sin(\kappa_{p}) & 0\\ \sin(\kappa_{p}) & \cos(\kappa_{p}) & 0\\ 0 & 0 & 1 \end{array}\right]}^{-1}\left[\begin{array}{c} r\\ \begin{array}{l} 0\\ \Delta h \end{array} \end{array}\right].$$

#### 2.2.3. Correcting Initial Transformation Parameters Using Iterative Minimum Entropy

As described in Section 2.1, the distance r between the scan positions of P and Q generally has an error up to 20 m. Search for the minimum entropy is not always the ideal case shown in Figure 4. In this paper, we propose a method called Iterative Minimum Entropy (IME) to correct r when searching for the minimum entropy and reduce the impact from smartphone GPS location error. The process of IME is as follows:

1 Initial distance and rotation angle detection

**Figure 5:**
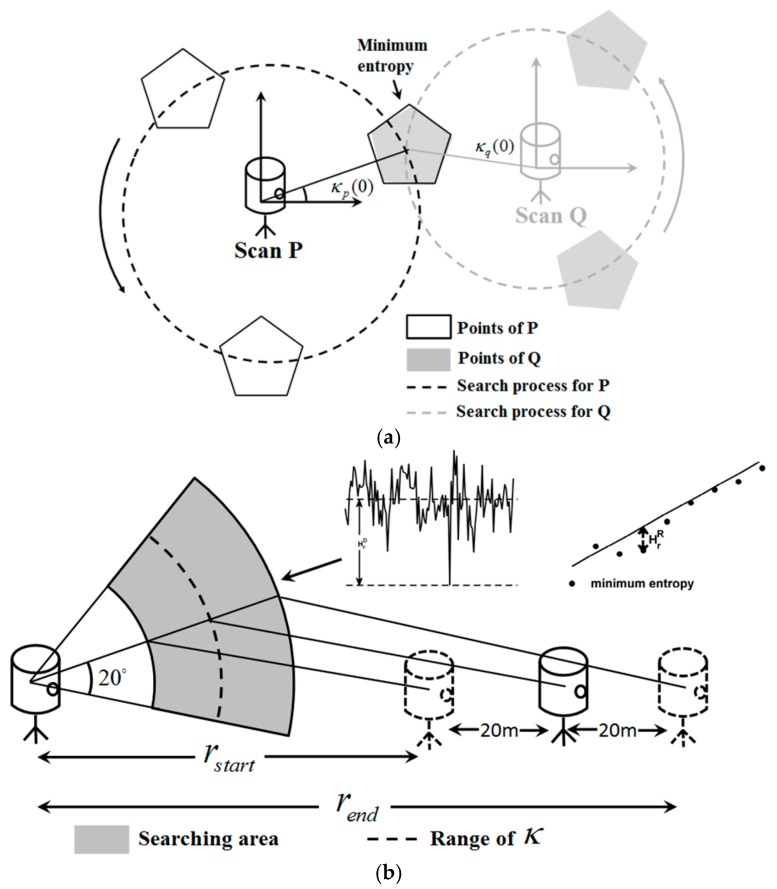
Search for the initial parameters: (**a**) the searching process for rotation angles; and (**b**) the searching process for initial scan distance.

The distance r between two adjacent scans (hereafter referred to as scan distance) can be directly calculated from scan position’s GPS coordinates. As shown in Figure 5a, the angles corresponding to the minimum entropy are selected as the initial rotation parameters,

(13) $$\left[\kappa_{p}(0),\kappa_{q}(0)]=\arg\;\min\;H_{r}(\kappa_{p},\kappa_{q}),\kappa_{p},\kappa_{q}\in [0,360^{\circ }\right].$$

The smartphone GPS generally has an error of up to 10 m, leading to a 20-m error in scan distance r. Thus, the true value of r commonly falls in the range of [r − 20, r + 20]. This range may be unreasonable; for example, r − 20 may be negative, or r +20 may be too large to conform to the actual situation. Here, we set two thresholds *r_low_* and *r_up_* to establish a further limit. The initial search range for scan distance can be obtained by

(14) $$r_{start}(0)=max\left(r_{low},r-20\right),$$

(15) $$r_{end}(0)=min\left(r_{up},r+20\right),$$

where $r_{low}$ is zero or a small value to ensure $r_{start}$ positive and $r_{\mathrm{up}}$ can be easily set to r+20 or a known value the scan distance will certainly not exceed.

The aim of the initial scan distance detection is to find the distance closest to the true value in [$r_{start}(0)$,$r_{end}(0)$]. Ten sampling values are selected to help the traversal, with the interval labeled as r_interval_,

(16) $$r_{k}=r_{start}(0)+\left(k-1\right)*r_{interval}(0),\;k=1,2\ldots 10,$$

(17) $$r_{interval}(0)=(r_{start}(0)-r_{end}(0))/9.$$

**Figure 6:**
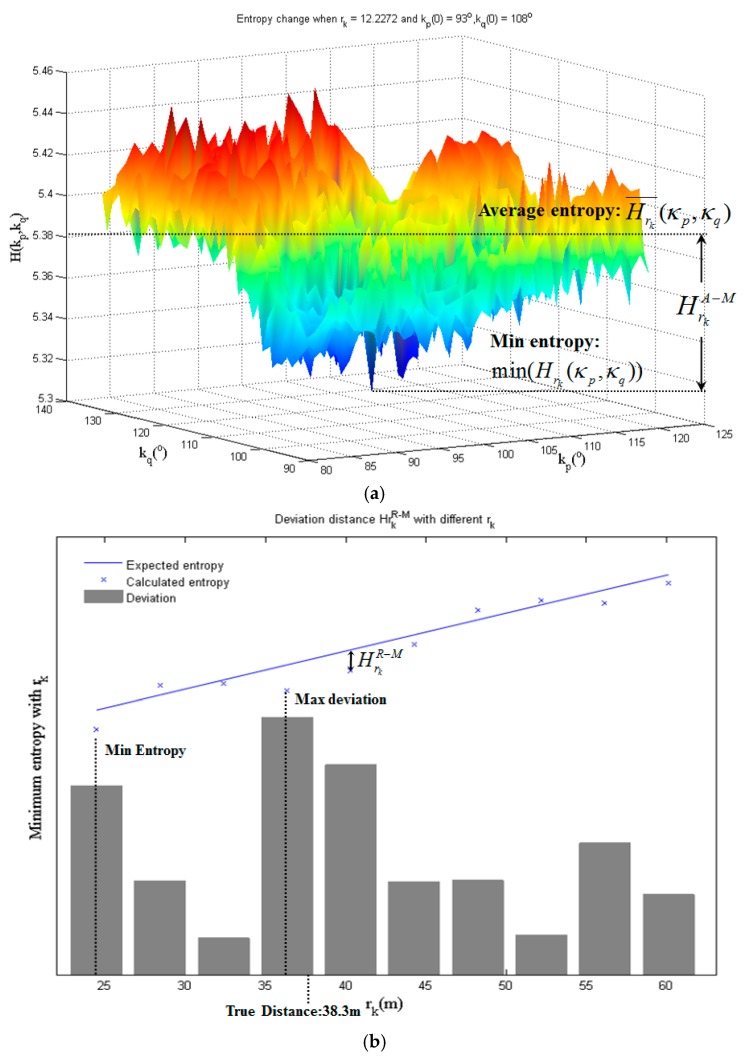
Two criteria for initial distance and rotation angles selection: (**a**) The entropy change when $r_{k}$ = 14.2272 m and $\kappa_{p}(0)$ = 93°, $\kappa_{q}(0)$ = 108°, in S1 and S2 of the first data set in Section 3.1. The calculation of $H_{r_{k}}^{A-M}$ corresponding to 12.2272 m is also shown. (**b**) How $H_{r_{k}}^{R-M}$ works on S3 and S4 in the second data set in Section 3.2.

For each *r_k_*, a series of entropy values are calculated within the range of $\kappa_{p}\in [\kappa_{p}(0)-20^{\circ },\kappa_{p}(0)+20^{\circ }],\kappa_{q}\in [\kappa_{q}(0)-20^{\circ },\kappa_{q}(0)+20^{\circ }]$, as shown in Figure 6a. With those entropy values, the difference between average and minimum entropy (Figure 6a) and the deviation from minimum entropy to expected entropy (Figure 6b), labeled as $H_{r_{k}}^{A-M}$ and $H_{r_{k}}^{R-M}$, are calculated to evaluate *r_k_*, as shown in Figure 5b. The criterion to select the initial scan distance is

(18) $$r(0)=\arg\min H_{r_{k}}^{R-M},r_{k}\in \{r_{k}|H_{r_{k}}^{A-M}>\bar{H_{r_{k}}^{A-M}},r_{k}\in [r_{start}(0),r_{end}(0)]\},$$

where $H_{r_{k}}^{A-M}$ is used to narrow the selection to some preliminary search results and larger $H_{r_{k}}^{A-M}$ commonly means more discriminative minimum entropy for *r_k_*. The $H_{r_{k}}^{A-M}$ is calculated by

(19) $$\begin{array}{l} H_{r_{k}}^{A-M}=\bar{H_{r_{k}}}(\kappa_{p},\kappa_{q})-min(H_{r_{k}}(\kappa_{p},\kappa_{q}))\\ \kappa_{p}\in [\kappa_{p}(0)-20^{\circ },\kappa_{p}(0)+20^{\circ }],\kappa_{q}\in [\kappa_{q}(0)-20^{\circ },\kappa_{q}(0)+20^{\circ }] \end{array},$$

where $\bar{H_{r_{k}}}(\kappa_{p},\kappa_{q})$ is the average entropy obtained within the preset angel range.

For $H_{r_{k}}^{R-M}$, we find that the entropy is partially affected by the scan distance between P and Q, that $H_{r_{k}}$ will generally become smaller with a smaller *r_k_*, and the effect can be ignored when it comes to a small variation range of *r*. Although smaller minimum entropy commonly means the corresponding *r_k_* closer to the true value, comparing the minimum entropy directly for different *r_k_* becomes unreliable here with the largest variation range of 40 m, as shown in Figure 6b. To eliminate the effect of distance, the series of entropy values $H_{r_{k}}(\kappa_{p}(0),\kappa_{q}(0))$ (*r_k_* sequence is obtained through Equations (16) and (17)) are selected as the reference and the relation between the entropy and distance is constructed based on linear model $H_{r_{k}}^{R}=m*r_{k}+b$. We prefer *r_k_* with the largest deviation from the minimum to reference entropy, instead of *r_k_* with the minimum entropy. The deviation $H_{r_{k}}^{R-M}$ is calculated by

(20) $$\begin{array}{l} H_{r_{k}}^{R-M}=min(H_{r_{k}}(\kappa_{p},\kappa_{q}))-H_{r_{k}}^{R},k=1,2\ldots 10,\\ \kappa_{p}\in [\kappa_{p}(0)-20^{\circ },\kappa_{p}(0)+20^{\circ }],\kappa_{q}\in [\kappa_{q}(0)-20^{\circ },\kappa_{q}(0)+20^{\circ }]. \end{array}$$

2 Iterative Minimum Entropy

With initial parameters obtained, the scan distance and rotation parameters will be corrected iteratively. In *i*-th iteration, the searching scope will be

(21) $$r_{start}(i)=max(r(i-1)-r_{interval}(i-1),r_{start}(0)),$$

(22) $$r_{end}(i)=min(r(i-1)+r_{interval}(i-1),r_{end}(0)),$$

(23) $$r_{interval}(i)=(r_{start}(i)-r_{end}(i))/9,i\geq 1,$$

where r(i−1) is the scan distance obtained after i − 1 times iteration and [$r_{start}(i)$,$r_{end}(i)$] will be the search region and $r_{\mathrm{int}erval}(i)$ be the search step. The *r_k_* will be traversed in [$r_{start}(i)$,$r_{end}(i)$] to find the optimal transformation parameters,

(24) $$\begin{array}{l} r(i)=\arg\;\min\;H_{r_{k}}(\kappa_{p},\kappa_{q}),r_{k}\in [r_{start}(i),r_{end}(i)],\\ {}[\kappa_{p}(i),\kappa_{q}(i)]=\arg\;\min\;H_{r(i)}(\kappa_{p},\kappa_{q}),\\ \kappa_{p}\in [\kappa_{p}(0)-20^{\circ },\kappa_{p}(0)+20^{\circ }],\kappa_{q}\in [\kappa_{q}(0)-20^{\circ },\kappa_{q}(0)+20^{\circ }], \end{array}$$

where the scan distance r(i) is selected with the minimum entropy in the iteration, because of the shrinking search scope, and the rotation parameters are calculated based on r(i).

3 Convergence

The change of the distribution entropy between two adjacent iterations is utilized as the condition of convergence. The algorithm stops when the entropy change is less than threshold τ, which is set 0.001 in this paper.

With the scan distance and rotation angles obtained at convergence, the coarse transformation parameters can be obtained by the Equation (12). The result of coarse alignment is used as the input for ICP in fine registration stage.

## 3. Experiments and Discussion

Two data sets obtained by a Reigl VZ-400 scanner were used to process and evaluate the proposed approach. Data set 1 was captured on a square, in which the primary objects are plants, sculptures and buildings around the square and covered a panoramic view. Data set 2 comprised scans captured from 6 positions around a building and only covered a horizontal view of about 100°. The primary target of data set 2 is a building with some plants around it.

All scans in one data set are aligned manually and refined by standard ICP to generate reference transformation parameters. Because IME is a method for coarse registration, the result of IME is processed with the ICP algorithm to achieve a complete registration. Errors between reference and estimated transformation parameters are used to estimate the registration accuracy. In addition, root mean square deviation (RMSD) before and after ICP is used here for metric accuracy and RMSD is calculated as

(25) $$RMSD=\sqrt{1/n\sum_{i=1}^{n}{\left(p_{i}-q_{i}\right)}^{2}},p_{i}\in P,q_{i}\in Q.$$

As described in Section 2.2.2, there are two parameters that must be set manually: d_G_ and t_G_. We also tested parameter sensitivity by changing one parameter while another remained unchanged.

### 3.1. Data Set 1

**Figure 7:**
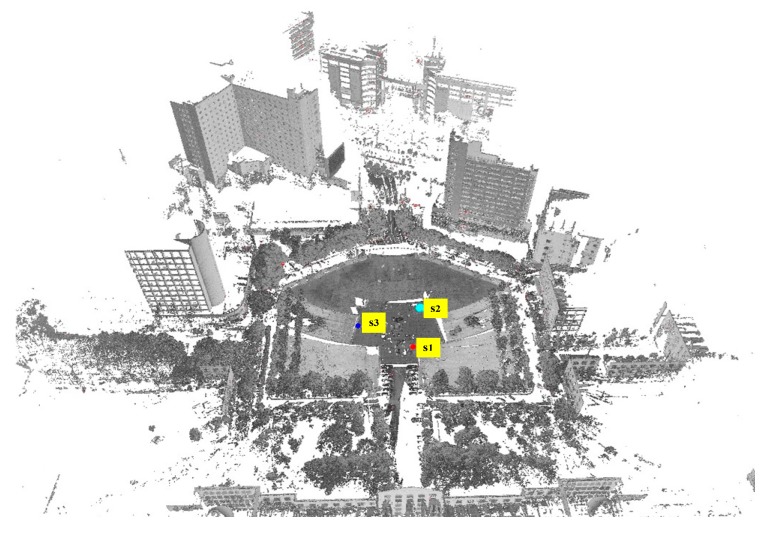
View of data set 1 after registration and three scan positions.

Data set 1 contains scans recorded at 3 different positions, as shown in Figure 7. Each scan in data set 1 contains approximately 50 million points spanning 360° horizontally and 100° vertically with a maximum range of approximately 400 m and dimensions of 537 m × 483 m × 111 m. The angular resolution is 0.02° and point density is between 8 and 80 mm. As illustrated in Figure 7, data set 1 contains a square in the middle with plants around it and buildings at a distance.

Table 1 shows the basic information of each scan, including the number of points in different stages. Block size d_G_ and t_G_ for point simplification and projection are both 1 m and the number of points is obviously decreased after projecting, improving the efficiency of IME significantly. Table 2 shows the distance between each scan position, as well as the corresponding reference distance, which is calculated from the result of manual alignment. Generally, there is a significant error for each scan distance that is unstable and cannot be removed as system error.

Table 3 shows the registration result, including rotation and translation errors, RMSD in IME and ICP and scan distance error ∆d_S_. For the search scope of the scan distance, r_low_ is set to 0, ensuring the distance is positive, and no upper limit is set (or r_up_ can be set to an extremely large value).

The rotation error is generally within 2°, with Δκ commonly smaller than the other two, because the rotation angle corresponding to the z axis is continuously corrected during IME iteration. Similarly, IME performs better on ∆z than the other two translation errors, demonstrating that it is feasible to directly use the distance between the ground parts along the z axis as the z component of translation for two adjacent scans. Although horizontal translation errors ∆x and ∆y are much larger than ∆z, the errors remain sufficient for a successful fine alignment, with translation errors and RMSE all reaching centimeter level after ICP. In addition, the error of scan distance also reaches centimeter or sub-centimeter level after ICP. In Table 3, we can also observe that the correlation between ∆d_S_ and transformation errors is not absolute, although in most cases, a smaller ∆d_S_ tends to correspond to smaller transformation errors. In conclusion, the IME algorithm is more concerned with the overall distribution of points in two adjacent scans, resulting in larger initial transformation errors than feature-based method in the stage of rough alignment, but the coarse alignment accuracy is still good enough to ensure the convergence of ICP in the fine alignment stage.

**Figure 8:**
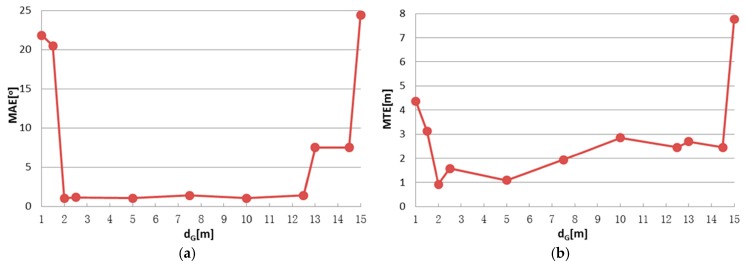

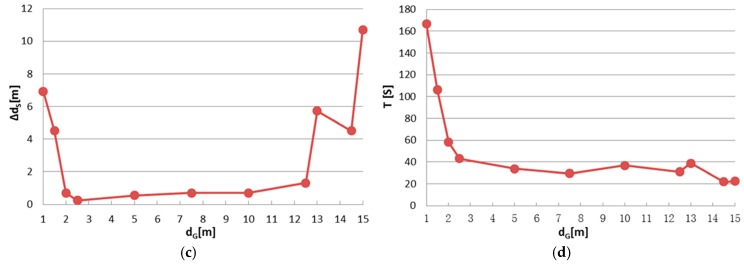
IME results with different d_G_, when t_G_ is fixed to 10 m: (**a**) mean angular error (MAE); (**b**) translation error (MTE); (**c**) scan distance error (∆d_S_); and (**d**) runtime (T).

**Figure 9:**
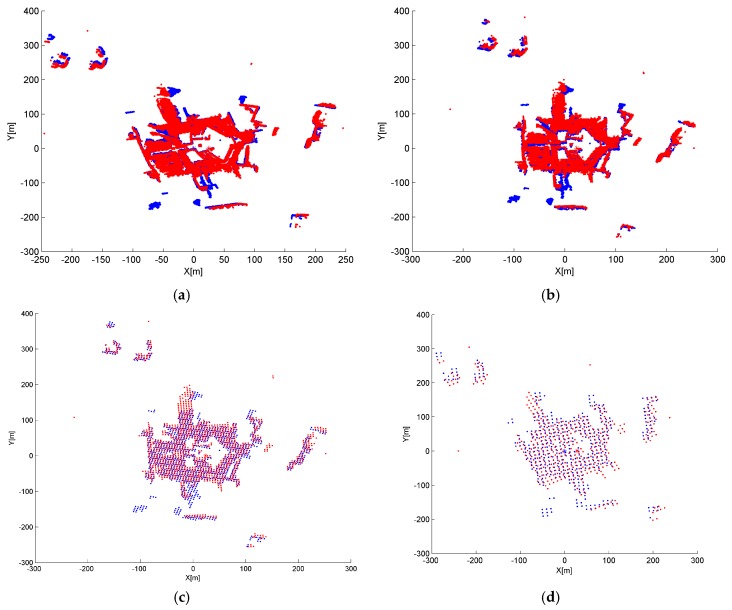
Nadir view of the spatial distributions for S1 and S2 after IME when: (**a**) d_G_ = 1 m; (**b**) d_G_ = 2.5 m; (**c**) d_G_ = 5 m; and (**d**) d_G_ = 10 m, and t_G_ is fixed to 10 m.

**Figure 10:**
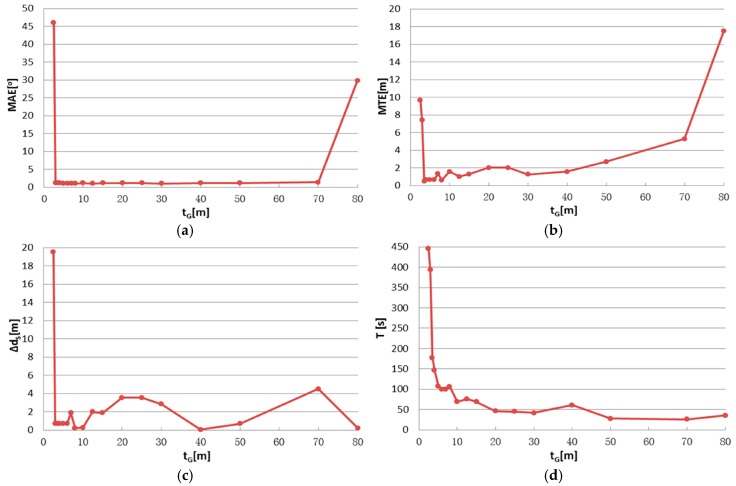
IME results with different t_G_, when d_G_ is fixed to 2.5 m: (**a**) mean angular error (MAE); (**b**) mean translation error (MTE); (**c**) scan distance error (∆d_S_); and (**d**) runtime (T).

**Figure 11:**
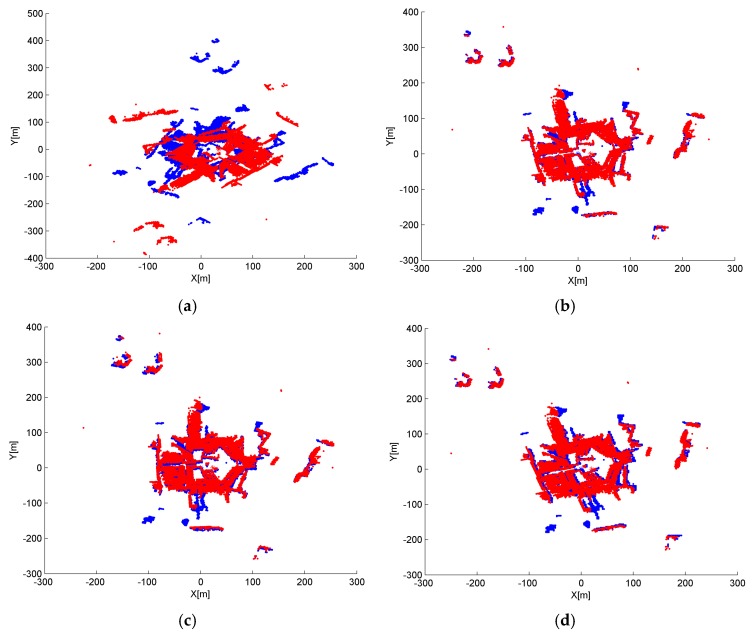
Nadir view of the spatial distributions after IME when: (**a**) t_G_ = 2.5 m; (**b**) t_G_ = 5 m; (**c**) t_G_ = 10 m; and (**d**) t_G_ = 20 m and d_G_ is fixed to 2.5 m.

There are two parameters in IME that have to be set by the user: block size d_G_ for the grid simplification of original points and block size t_G_ for entropy calculation, as described in Section 2.2.2. To identify the appropriate parameter settings and test the parameter stability, registration tests were conducted using different parameter settings. Test results are shown in Figure 8, Figure 9, Figure 10 and Figure 11, in which the mean angular error (MAE), mean translation error (MTE), scan distance error (∆d_S_) and runtime T are analyzed. In the test, MAE and MTE are only influenced by Δκ and ∆x, ∆y, respectively, because ∆φ, Δω and ∆z are all set to fixed values during IME iteration.

Although a smaller grid size d_G_ or t_G_ can retain more details from the original point cloud, we observed that incorrect transformation parameters are obtained with an MAE greater than 20° when d_G_ is below 2 m or t_G_ is below 3 m in Figure 8 and Figure 10. The ICP can obtain a right convergence when d_G_ is between 2 m and 14.5 m in Figure 8. Considering the runtime and translation errors, [2.5, 7.5] will be a more appropriate variation range for d_G_. Figure 9 shows the projections of four rough alignment results when d_G_ is 1 m, 2 m, 5 m and 10 m. By comparing Figure 9b,c, we observe that despite having similar angular and translation errors, slight differences remain in the results of rough alignment. IME performs better on the two buildings located in the upper left corner when d_G_ is 5 m whereas the parameter setting d_G_ = 2.5 m gains a greater overlap on the building located in the lower right corner. The best results are achieved when d_G_ is approximately 50 to 100 times the average density and the stability of the rotation error is better than that of the translation error.

Figure 10 and Figure 11 show similar tendency with Figure 8 and Figure 9; thus, similar conclusions can be drawn. MAE is below 2 m when t_G_ is in the range of [3,40], which is much wider than the that of d_G_. We also note that t_G_ has more influence on the efficiency than d_G_, by comparing Figure 8d and Figure 10d. The best results are achieved when t_G_ is approximately 1% to 10% of the shortest edge of the point cloud’s bounding box. In addition, t_G_ has better error resistance than d_G_ while the latter has better runtime stability.

### 3.2. Data Set 2

**Figure 12:**
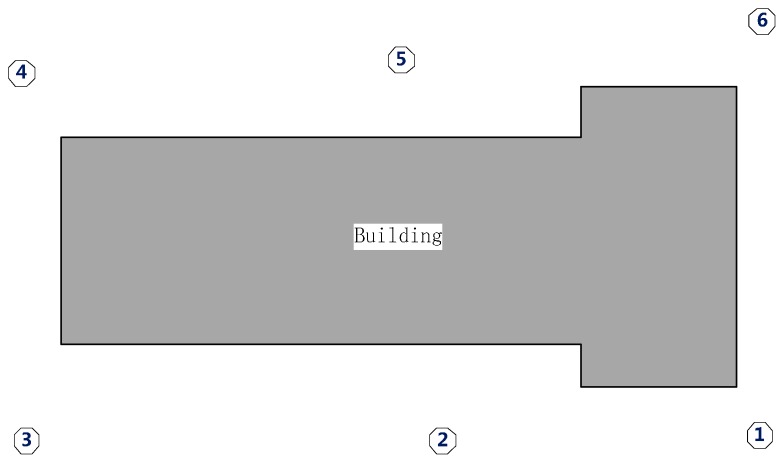
Distribution of the 6 scans.

Compared with data set 1, the horizontal scanning scope of data set 2 (as shown in Figure 12) is between 60 and 100° with a lower overlap rate. Data set 2 comprises 6 scans around a building with surroundings including vegetation, roads, cars and bicycles and was acquired with dimensions ≈ 85 m × 166 m × 49 m and point density between 8 and 16 mm. The basic information is shown in Table 4, and Table 5 shows the distance between different scan pairs in data set 2. Scan pairs S1–S2 and S5–S6 have a deviation of more than 15 m from the reference values. This is because a large amount of vegetation interfered with the GPS signal when the scans were acquired.

**Figure 13:**
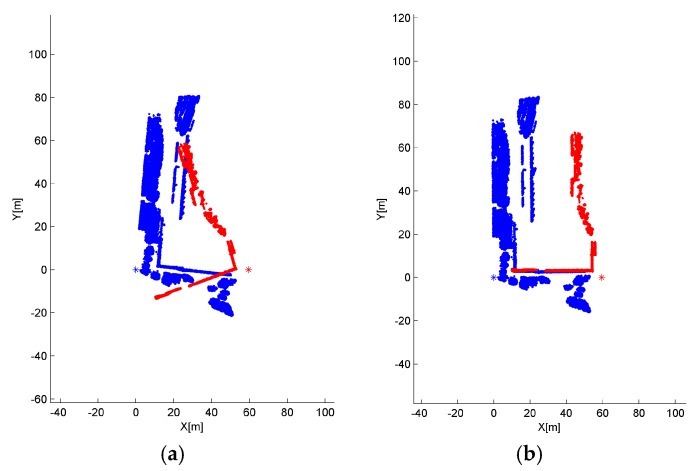
The IME result of S1–S6: (**a**) the Nadir view corresponding to the minimum entropy, which is a local convergence for IME; and (**b**) the theoretically correct posture for S1 and S2, which corresponds to the fifth small entropy.

The registration test was conducted with parameter settings: d_G_ = 0.15 m and t_G_ = 2.5 m, recalling the conclusion of the test on dataset 1. The result is shown in Table 6, with the overlap rate of neighboring scans. The registration error of data set 2 is larger than that of data set 1 and the likely reason is the lower overlap rate between adjacent scans, resulting in the decreased accuracy of initial transformation parameters. Despite this, most results of IME remains good enough to be correctly refined by ICP in the stage of fine alignment, with the final accuracy on the order of scanner’s measurement accuracy, except the registration between S1 and S6. As shown in Table 6 and Figure 13, the IME falls into the local convergence basin for the registration of S1 and S6, probably because of the small overlap rate and the homogeneous overlapping area, containing only part of a wall and placing weak constraint on the transformation search. We also tried to use the result in Figure 13b as the input of ICP, but the z component of the translation parameters still contained a large error, for there is little overlap at the ground part. For the two nonadjacent scan pairs, S1–S3 and S4–S6, although the overlap rates are significantly lower compared with the sequential pairs, the registration results are still satisfying, with the registration errors at centimeter level.

**Figure 14:**
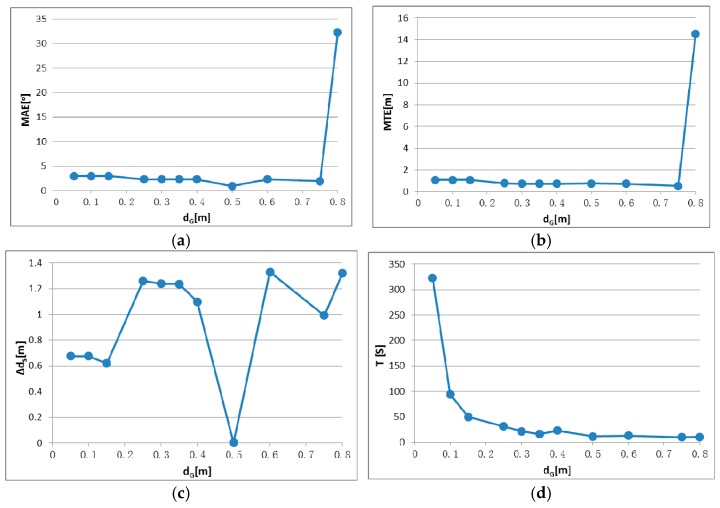
IME results with different d_G_, when t_G_ is fixed to 2.5 m: (**a**) mean angular error (MAE); (**b**) mean translation error (MTE); (**c**) scan distance error (∆d_S_); and (**d**) runtime (T).

**Figure 15:**
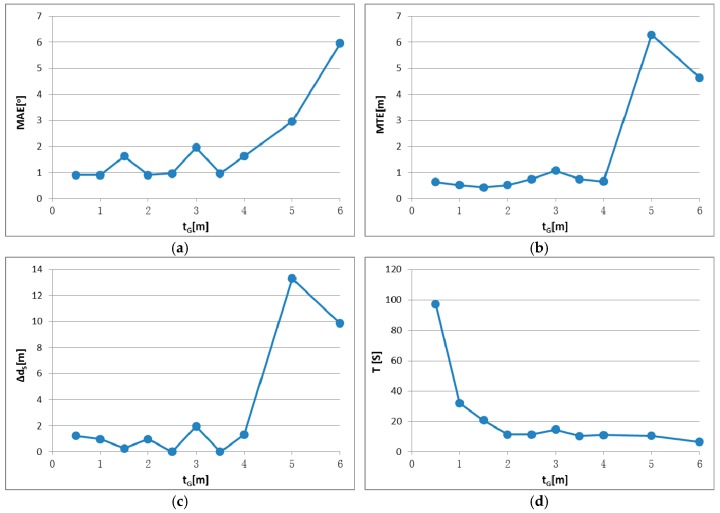
IME results with different t_G_ when d_G_ is fixed to 0.5 m: (**a**) mean angular error (MAE); (**b**) mean translation error (MTE); (**c**) scan distance error (∆d_S_); and (**d**) runtime (T).

The results under different d_G_ and t_G_ settings are shown in Figure 14 and Figure 15. The experiment on data set 2 shows similar trends with data set 1, except for the correct result under the minimum parameter setting, d_G_ = 0.05 m and t_G_ = 0.05 m. Considering the error and runtime together, the best results are obtained when d_G_ is between 0.05 m and 0.75 m, and t_G_ is between 0.05 m and 4 m and the conclusion can be drawn similar as that of data set 1.

The accuracies shown in Figure 14 and Figure 15 are slightly worse than in Figure 8 and Figure 10 and the range of d_G_ and t_G_ corresponding to correct results is smaller. This result is most likely because of the smaller dimensions of data set 2. Two distant points may fall into the same block if d_G_ or t_G_ is set too large compared with the edge length of the bounding box.

**Figure 16:**
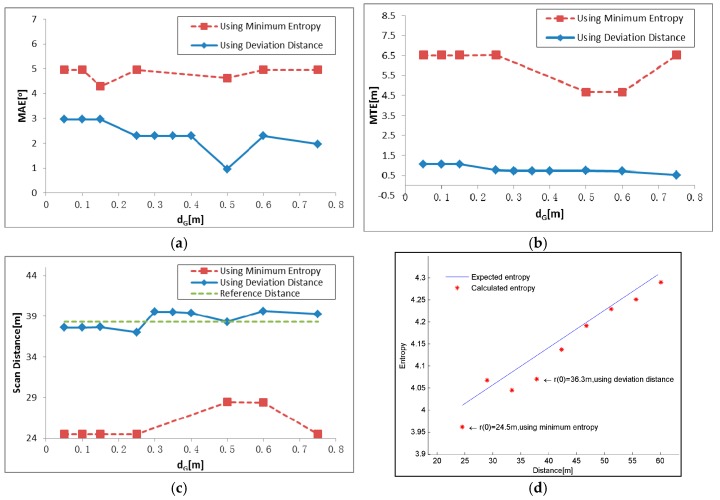
Comparison of two initial value selection criteria, using minimum entropy and using deviation distance: (**a**) MAE; (**b**) MTE; and (**c**) Scan distance; and (**d**) the selecting process in detail. Although the distance 24.5 m corresponds to the smallest entropy, 36.3 m, which is much closer to reference distance of 38.302 m, is selected based on deviation distance.

In addition to parameter tests, we conducted a further test to compare two initial value selecting criteria: using minimum entropy and deviation distance. This test was conducted using parameter settings: *d_G_* = 0.5 m and *t_G_* = 0.25 m, and the result is shown in Figure 16. As shown in Figure 16a–c, the results based on the deviation show a better performance, with smaller MAE and MTE and the scan distance much closer to the reference. Figure 16d shows the selecting process of scan distance. The linear function between entropy and distance is shown in the form of line, and ten candidates are labeled as points. The 2D distribution entropy shows a decreasing trend when the scan distance reduces. The minimum distance 24.5 m will be selected as the initial scan distance if the minimum entropy is used as decision criterion, as shown in Figure 16d. When the deviation is used as criterion, the initial scan distance will be 36.3 m, which is much closer to the reference scan distance 38.302 m, erasing the entropy change brought by distance variation.

### 3.3. Comparison

**Figure 17:**
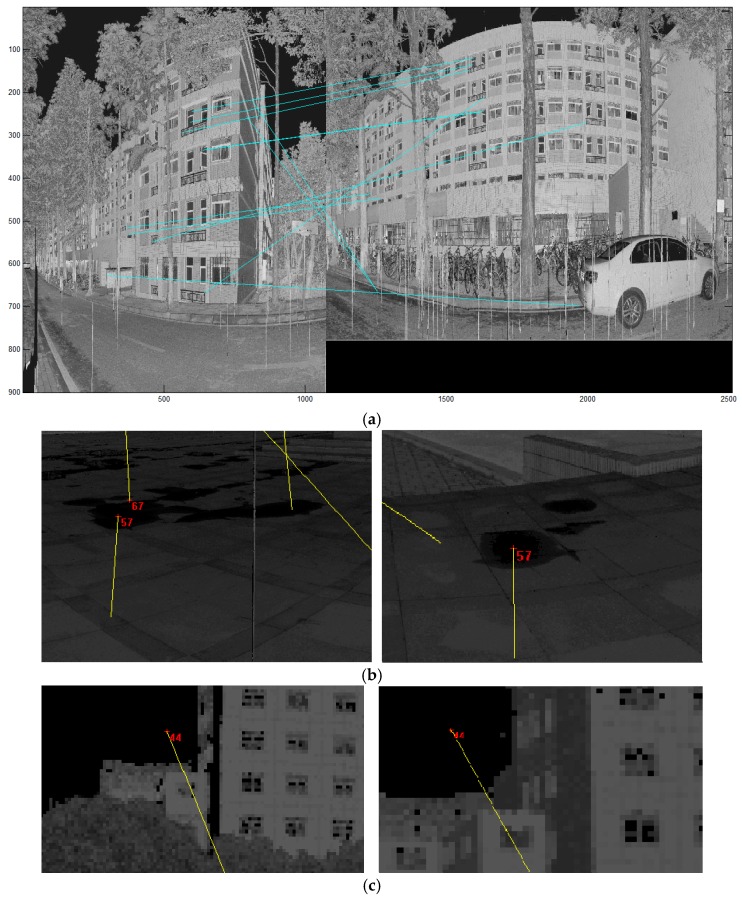
Matching results using SIFT: (**a**) the result of SIFT feature matching on S1–S2 in data set 2 and the false matching is mainly because of viewpoint changes and self-similarity; (**b**) the false match from holes; and (**c**) the correct match on the background with no correspondence in the space of point cloud.

A comparison was taken between SIFT-based method and the proposed method. In the SIFT-based method, correspondences are obtained on the reflectance image with SIFT and false matches are excluded with the branch-and-bound algorithm [19,23]. The mean angle error, mean translation error and RMSD before ICP are listed in Table 7. As shown in Figure 17, the matching rate is limited because of the viewpoint changes, self-similarity and holes, and some problems may exist, e.g., the points on background and inaccurate matching. Meanwhile, the proposed method, which is independent of detailed features, mainly focuses on the overall distribution and reduces the possibility of false registration result.

## 4. Conclusions

In this paper, a method called Iterative Minimum Entropy (IME) is proposed for the coarse registration of TLS point clouds, with a novel sensor combination mode of terrestrial laser scanner and smartphone. This method is based on 2D distribution entropy and the distance r between neighboring scan positions, by rotating two point clouds around their own z axis until the optimal initial transformation is reached. Since there is no synchronization between the laser scanner and smartphone, only a rough distance between neighboring scanner positions is measured using smartphone GPS. We proposed two criteria, the difference between average and minimum entropy and the deviation from minimum entropy to expected entropy, to decide the optimal initial transformation between two scans instead of directly using the minimum entropy. The method achieved high accuracy and efficiency in the two experiments we have conducted, in which panoramic and non-panoramic, vegetation-dominated and building-dominated scenes were tested. Commonly, the proposed method achieves a good result when t_G_ is approximately 1% to 10% of the shortest edge of the bounding box and d_G_ is approximately 50 to 100 times the average density, according to the experimental results, but the range is not absolute. It is also noticed that the IME is likely to fall into a local convergence basin when the overlapping rate is too low or most overlapping areas are of narrow distribution.

The future investigations will include efforts to deal with the small overlapping rate, which is about 5%–10%, and homogeneous overlapping areas. In addition, IME can be extended in the future using other ranging methods, e.g., Electronic Distance Measurement (EDM), or the initial distance can simply be given by the user in some special cases, such as near the subway or train tracks, where each track section has a fixed length and can be treated as the distance marker, making it a method using no prior information. However, some modifications may be needed to ensure the robustness for different manners of extension.

## Figures and Tables

**Table 1:** Basic information of each scan.

Scan	Number of Points		
	Original	After Preprocessing	After Projecting
S1	50,164,568	21,225,851	15,017
S2	53,101,629	21,256,225	15,749
S3	54,158,850	21,858,623	15,631

**Table 2:** Initial distance between adjacent scan positions and the corresponding reference.

Scan pair	Scan Distance (m)	Reference (m)
S1–S2	34.2272	24.5040
S1–S3	25.8609	28.264
S2–S3	40.4741	31.6358

**Table 3:** Errors between estimated and reference transformation parameters: ∆φ, Δω and Δκ are rotation parameter errors corresponding to the x, y and z axes, respectively; ∆x, Δy and Δz are translation errors along the x, y and z axes, respectively; RMSD is the root mean square deviation between two point clouds; and ∆d_S_ is the error of the distance between two adjacent scan positions.

Scans	Stage	∆φ (°)	Δω (°)	Δκ (°)	∆x (m)	∆y (m)	∆z (m)	RMSD (m)	∆d_S_ (m)
S1–S2	IME	0.317	2.501	−0.639	3.784	0.644	0.433	0.643	0.220
	ICP	0.034	0.173	0.020	0.162	0.081	−0.006	0.011	−0.063
S1–S3	IME	−1.011	0.316	0.171	−1.065	1.482	−0.358	0.517	−1.292
	ICP	−0.008	0.010	−0.003	−0.006	0.004	−0.006	0.007	−0.007
S2–S3	IME	−1.986	−1.613	−1.128	−2.665	−0.608	−0.281	0.853	−2.27
	ICP	−0.143	−0.059	0.035	−0.07	−0.031	−0.044	0.007	−0.076

**Table 4:** Basic information of each scan.

Scan	Number of Points		
	Original	After Preprocessing	After Projecting
S1	16,941,512	14,168,335	31,652
S2	20,841,940	12,642,348	11,736
S3	10,091,225	7,832,464	15,022
S4	14,232,022	10,745,991	7119
S5	22,715,235	14,635,858	9299
S6	16,474,111	10,901,182	4819

**Table 5:** Initial distance of each scan pair and the corresponding reference.

	Distance (m)	S1	S2	S3	S4	S5	S6
Reference (m)							
S1		0	43.4	84.4	98.5	53.1	59.5
S2		28.712	0	43	69.6	37.9	72.7
S3		74.967	46.529	0	44.5	48.5	90.6
S4		88.723	65.984	38.302	0	54.4	76
S5		63.112	56.282	63.939	47.843	0	48.7
S6		58.443	65.705	90.439	77.565	28.896	0

**Table 6:** Errors between estimated and reference transformation parameters.

Scan Pair	Overlap Rate	Stage	∆φ (°)	Δω (°)	Δκ (°)	∆x (m)	∆y (m)	∆z (m)	RMSD (m)	∆d_S_ (m)
S1–S2	17.3%	IME	2.23	1.396	0.230	0.133	0.798	0.609	0.25	0.795
		ICP	0.205	0.402	−0.029	0.024	0.039	0.005	0.018	0.045
S1–S3	10.84%	IME	1.192	1.691	−2.865	0.749	−0.998	0.186	0.480	0.567
		ICP	0.422	0.545	0.329	0.302	−0.244	0.075	0.039	−0.078
S1–S6	7.67%	IME	-	-	-	-	-	-	-	-
		ICP	-	-	-	-	-	-	-	-
S2–S3	23.5%	IME	−1.038	0.295	−5.895	5.236	1.404	−0.423	0.58	1.963
		ICP	−0.246	0.145	−0.131	0.028	0.109	0.0797	0.028	0.112
S3–S4	17.8%	IME	−0.466	1.330	6.090	−2.170	−0.922	0.111	0.103	0.983
		ICP	0.304	−0.382	−0.047	0.126	−0.109	0.108	0.012	−0.167
S4–S5	18.1%	IME	−0.313	−1.940	−2.115	−0.371	−1.641	0.671	0.523	−0.733
		ICP	0.087	0.085	−0.144	0.0379	−0.114	0.0004	0.021	0.013
S4–S6	10.52%	IME	0.140	−1.10	1.091	−0.182	0.433	−0.059	0.140	−0.096
		ICP	−0.091	0.011	−0.004	0.079	−0.002	−0.029	0.037	0.077
S5–S6	30.1%	IME	−0.551	−0.779	−4.011	−1.148	1.134	−0.730	0.231	0.820
		ICP	0.027	0.0054	0.0005	0.017	−0.002	−0.022	0.011	0.102

**Table 7:** Comparison between the proposed method and SIFT-based method.

	SIFT Based Method						The Proposed Method		
	Key Points	Average Match	Successful Match (%)	Average MAE (°)	Average MTE (m)	Average RMSD (m)	Average MAE (°)	Average MTE (m)	Average RMSD (m)
Data set 1	3678	72	69.4	1.741	1.09	0.715	1.076	1.258	0.671
Data set 2	7608	5	-	-	-	-	1.532	0.958	0.330

## References

[1] Franaszek M., Cheok G.S., Witzgall C. (2009). Fast automatic registration of range images from 3D imaging systems using sphere targets. Autom. Constr..

[2] Akca D. (2003). Full Automatic Registration of Laser Scanner Point Clouds.

[3] Besl P., McKay N.D. (1992). A method for registration of 3-D shapes. IEEE Trans. Pattern Anal. Mach. Intell..

[4] Chen Y., Medioni G. (1992). Object modelling by registration of multiple range images. Image Vis. Comput..

[5] Gruen A., Akca D. (2005). Least squares 3D surface and curve matching. ISPRS J. Photogramm. Remote Sens..

[6] Pottmann H., Huang Q.X., Yang Y.L., Hu S.M. (2006). Geometry and Convergence Analysis of Algorithms for Registration of 3D Shapes. Int. J. Comput. Vis..

[7] Rabbani T., Dijkman S., van den Heuvel F., Vosselman G. (2007). An integrated approach for modelling and global registration of point clouds. ISPRS J. Photogramm. Remote Sens..

[8] Censi A. An ICP variant using a point-to-line metric. Proceedings of the IEEE International Conference on Robotics and Automation (ICRA).

[9] Gressin A., Mallet C., Demantké J., David N. (2012). Towards 3D lidar point cloud registration improvement using optimal neighborhood knowledge. ISPRS J. Photogramm. Remote Sens..

[10] Lowe D.G. (2004). Distinctive image features from scale-invariant keypoints. Int. J. Comput. Vis..

[11] Bendels G.H., Degener P., Wahl R., Körtgen M., Klein R. Image-based registration of 3D-range data using feature surface elements. Proceedings of the International Conference on Virtual Reality, Archaeology and Intelligent Cultural Heritage, Eurographics Association.

[12] Barnea S., Filin S. (2008). Keypoint based autonomous registration of terrestrial laser point-clouds. ISPRS J. Photogramm. Remote Sens..

[13] Böhm J., Becker S. Automatic marker-free registration of terrestrial laser scans using reflectance. Proceedings of the Proceedings of 8th Conference on Optical 3D Measurement Techniques.

[14] Wang Z., Brenner C. (2008). Point based registration of terrestrial laser data using intensity and geometry features. Int. Arch. Photogramm. Remote Sens. Spat. Inf. Sci..

[15] Kang Z., Li J., Zhang L., Zhao Q., Zlatanova S. (2009). Automatic registration of terrestrial laser scanning point clouds using panoramic reflectance images. Sensors.

[16] Kang Z. Automatic registration of terrestrial point clouds based on panoramic reflectance images and efficient BaySAC. Proceedings of the Eighth International Symposium on Multispectral Image Processing and Pattern Recognition, International Society for Optics and Photonics.

[17] Weinmann M., Weinmann M., Hinz S., Jutzi B. (2011). Fast and automatic image-based registration of TLS data. ISPRS J. Photogramm. Remote Sens..

[18] Kang Z., Zlatanova S., Gorte B. (2007). Automatic Registration of Terrestrial Scanning Data Based on Registered Imagery.

[19] Renaudin E., Habib A., Kersting A.P. (2011). Feature-Based Registration of Terrestrial Laser Scans with Minimum Overlap Using Photogrammetric Data. ETRI J..

[20] Canaz S., Habib A. (2013). Photogrammetric features for the registration of terrestrial laser scans with minimum overlap. J. Geod. Geoinf..

[21] Gelfand N., Mitra N.J., Guibas L.J., Pottmann H. Robust global registration. Proceedings of the Symposium on Geometry Processing.

[22] Theiler P.W., Wegner J.D., Schindler K. (2014). Keypoint-based 4-Points Congruent Sets–Automated marker-less registration of laser scans. ISPRS J. Photogramm. Remote Sens..

[23] Rusu R.B., Cousins S. 3D is here: Point cloud library (PCL). Proceedings of the 2011 IEEE International Conference on Robotics and Automation (ICRA).

[24] Aiger D., Mitra N.J., Cohen-Or D. (2008). 4-points congruent sets for robust pairwise surface registration. ACM Trans. Graph..

[25] Rusu R.B., Blodow N., Marton Z.C., Beetz M. Aligning point cloud views using persistent feature histograms. Proceedings of the 2008 IEEE/RSJ International Conference on Intelligent Robots and Systems.

[26] Rusu R.B., Blodow N., Beetz M. Fast point feature histograms (FPFH) for 3D registration. Proceedings of the IEEE International Conference on Robotics and Automation (ICRA’09).

[27] Liu R., Hirzinger G. (2005). Marker-Free Automatic Matching of Range Data.

[28] Basdogan C., Oztireli A.C. (2008). A new feature-based method for robust and efficient rigid-body registration of overlapping point clouds. Vis. Comput..

[29] Lo T.W.R., Siebert J.P. (2009). Local feature extraction and matching on range images: 2.5 D SIFT. Comput. Vis. Image Underst..

[30] Ge B., Peng B., Tian Q. (2013). Registration of three-dimensional point-cloud data based on curvature map. J. Tianjin Univ..

[31] Guo Y., Sohel F., Bennamoun M., Wan J., Lu M. (2014). An accurate and robust range image registration algorithm for 3D object modeling. IEEE Trans. Multimedia.

[32] Stamos I., Leordeanu M. Automated feature-based range registration of urban scenes of large scale. Proceedings of the IEEE Computer Society Conference on Computer Vision and Pattern Recognition.

[33] Yao J., Ruggeri M.R., Taddei P., Sequeira V. (2010). Automatic scan registration using 3D linear and planar features. 3D Res..

[34] Yang B., Zang Y. (2014). Automated registration of dense terrestrial laser-scanning point clouds using curves. ISPRS J. Photogramm. Remote Sens..

[35] Theiler P.W., Schindler K. (2012). Automatic registration of terrestrial laser scanner point clouds using natural planar surfaces. ISPRS Ann. Photogr. Remote Sens. Spat. Inf. Sci..

[36] Dold C., Brenner C. (2006). Registration of terrestrial laser scanning data using planar patches and image data. Int. Arch. Photogramm. Remote Sens. Spat. Inf. Sci..

[37] Brenner C., Dold C., Ripperda N. (2008). Coarse orientation of terrestrial laser scans in urban environments. ISPRS J. Photogramm. Remote Sens..

[38] Pu S., Li J., Guo S. (2014). Registration of Terrestrial Laser Point Clouds by Fusing Semantic Features and GPS Positions. Acta Geod. Cartogr. Sin..

[39] Yang B., Dong Z., Liang F., Liu Y. (2016). Automatic registration of large-scale urban scene point clouds based on semantic feature points. ISPRS J. Photogramm. Remote Sens..

[40] Kelbe D., Aardt J.V., Romanczyk P., van Leeuwen M., Cawse-Nicholson K. (2016). Marker-Free Registration of Forest Terrestrial Laser Scanner Data Pairs With Embedded Confidence Metrics. IEEE Trans. Geosci. Remote Sens..

[41] Yang B., Dai W., Dong Z., Liu Y. (2016). Automatic Forest Mapping at Individual Tree Levels from Terrestrial Laser Scanning Point Clouds with a Hierarchical Minimum Cut Method. Remote Sens..

[42] Wen X., Xu S., Elberink S.O., Vosselman G. (2016). Individual Tree Crown Modeling and Change Detection from Airborne Lidar Data. IEEE J. Sel. Top. Appl. Earth Obs. Remote Sens..

[43] Zeisl B., Koser K., Pollefeys M. Automatic registration of RGB-D scans via salient directions. Proceedings of the IEEE International Conference on Computer Vision.

[44] Chan T.O. (2015). Cylindrical and Polygonal Object Modelling and its use in LiDAR Calibration and Point Cloud Registration. Ph.D. Thesis.

[45] Rabbani T., van den Heuvel F. Automatic point cloud registration using constrained search for corresponding objects. Proceedings of the 7th Conference on Optical.

[46] Urban S., Weinmann M. (2015). Finding a good feature detector-descriptor combination for the 2D keypoint-based registration of TLS point clouds. ISPRS Ann. Photogramm. Remote Sens. Spat. Inf. Sci..

[47] Talaya J., Alamus R., Bosch E., Serra A., Kornus W., Baron A. Integration of a terrestrial laser scanner with GPS/IMU orientation sensors. Proceedings of the XXth ISPRS Congress.

[48] Kukko A., Kaartinen H., Hyyppä J., Chen Y. (2012). Multiplatform mobile laser scanning: Usability and performance. Sensors.

[49] Asai T., Kanbara M., Yokoya N. 3D modeling of outdoor environments by integrating omnidirectional range and color images. Proceedings of the Fifth International Conference on 3-D Digital Imaging and Modeling (3DIM’05).

[50] Sirmacek B., Lindenbergh R.C., Menenti M. Automatic registration of Iphone images to LASER point clouds of the urban structures using shape features. Proceedings of the ISPRS Annals of the Photogrammetry, Remote Sensing and Spatial Information Sciences.

[51] Zhang S., Shan J., Zhang Z., Yan J., Hou Y. Integrating Smartphone Images and Airborne LIDAR Data for Complete Urban Building Modelling. Proceedings of the ISPRS-International Archives of the Photogrammetry, Remote Sensing and Spatial Information Sciences.

[52] Shannon C.E. (2001). A mathematical theory of communication. ACM SIGMOBILE Mob. Comput. Commun. Rev..

[53] Jiao W., Long T., Yang G., He G. (2014). A New Method for Geometric Quality Evaluation of Remote Sensing Image Based on Information Entropy. Int. Arch. Photogramm. Remote Sens. Spat. Inf. Sci..

[54] Samanta D., Sanyal G. (2012). Classification of SAR Images Based on Entropy. Int. J. Inf. Technol. Comput. Sci..

[55] Pickering M.R., Jia X. Registration of multi-sensor remote sensing imagery by gradient-based optimization of cross-cumulative residual entropy. Proceedings of the SPIE—The International Society for Optical Engineering.

[56] Pirotti F., Guarnieri A., Vettore A. (2013). Ground filtering and vegetation mapping using multi-return terrestrial laser scanning. ISPRS J. Photogramm. Remote Sens..

[57] Vosselman G., Maas H.G. (2010). Airborne and Terrestrial Laser Scanning.

[58] Polewskia P., Ericksonc A., Yaoa W., Coopsc N., Krzysteka P., Stillab U. Object-Based Coregistration of Terrestrial Photogrammetric and ALS Point Clouds in Forested Areas. Proceedings of the ISPRS Annals of Photogrammetry, Remote Sensing and Spatial Information Sciences.

